# Pilot Analysis of Late Conversion to Belatacept in Kidney Transplant Recipients for Biopsy-Proven Chronic Tacrolimus Toxicity

**DOI:** 10.1155/2018/1968029

**Published:** 2018-05-02

**Authors:** Shruti Gupta, Ivy Rosales, David Wojciechowski

**Affiliations:** Massachusetts General Hospital, Boston, MA, USA

## Abstract

**Background:**

Calcineurin inhibitors are associated with chronic nephrotoxicity, manifesting as interstitial fibrosis/tubular atrophy (IF/TA) and arteriolar hyalinosis. Conversion from tacrolimus to belatacept may be one strategy to preserve renal function.

**Methods:**

We conducted a retrospective review of renal transplant patients followed at our institution who were converted to belatacept and found to have chronic tacrolimus toxicity on biopsy. The primary outcome was eGFR at conversion as compared to eGFR at 3, 6, 12, and 24 months after conversion. We also assessed incidence of infection and rates of allograft survival at 1 year.

**Results:**

The average time between transplant and conversion was 11.9 years. There was no decrease in eGFR at any postconversion time point as compared with preconversion. The mean eGFR at time of preconversion was 32.9 mL/min, as compared with 35.6 mL/min at 3 months (*p* = 0.09), 34.1 mL/min at 6 months (*p* = 0.63), 34.9 mL/min at 12 months (*p* = 0.57), and 39.6 mL/min at 24 months after conversion (*p* = 0.92). Four of 7 patients had increases in their eGFR after conversion. All grafts were functioning at 1 year after conversion.

**Conclusion:**

While this study was limited by a small number of patients, belatacept conversion stabilized eGFR at all time points in patients with late allograft function due to chronic tacrolimus toxicity, with a trend towards increased eGFR at 3 months.

## 1. Introduction

The 5-year graft survival rates for living donor and deceased donor transplants are at a disappointing 79.8% and 66.6%, respectively, in spite of a reduction in early acute rejection rates with more potent immunosuppression [1]. The current standard of care is to combine a calcineurin inhibitor (CNI) with an antimetabolite, with or without corticosteroids. The negative effects of CNIs are well documented and include an increased risk of cardiovascular disease, hypertension, and posttransplant diabetes [2, 3]. CNI nephrotoxicity can manifest as interstitial fibrosis/tubular atrophy (IF/TA) and arteriolar hyalinosis. CNIs are also less effective at preventing chronic alloantibody responses than they are at limiting acute T cell mediated responses, leading to a risk of donor specific antibody (DSA) development and chronic antibody mediated rejection [4]. Focus is now shifting to optimizing CNI-free immunosuppression regimens using belatacept. Seven-year outcomes from the BENEFIT study (Belatacept Evaluation of Nephroprotection and Efficacy as First-Line Immunosuppression Trial) showed that, among renal transplant patients who received regimens incorporating belatacept, the mean estimated glomerular filtration rate (eGFR) increased over 84 months as compared to the cyclosporine group, in whom the eGFR declined [5].

Though there are studies describing the benefits of early conversion to belatacept, few have evaluated the effects of late conversion and the potential impact of a maintenance immunosuppression regimen on patients experiencing renal allograft function decline with long-term CNI use and toxicity [6–8]. We hypothesized that, among patients with declining renal function and biopsy-proven chronic CNI toxicity, late conversion to belatacept would lead to stabilization of eGFR and improved allograft outcome.

## 2. Materials and Methods

We conducted a retrospective review of all renal transplant recipients followed at our center between 1994 and 2015. We identified patients who were converted to belatacept from tacrolimus due to evidence of chronic tacrolimus toxicity found on a renal biopsy performed* prior* to conversion. Biopsies were performed for a rising creatinine. Chronic tacrolimus toxicity was defined as histologic evidence of CNI toxicity (IF/TA and arteriolar hyalinosis by Banff classification) and worsening allograft dysfunction [9, 10]. Patients were excluded if they had findings other than chronic CNI toxicity or evidence of rejection. Patients who were started on belatacept de novo or who were converted within 5 years of transplant were excluded in order to select for those with chronic CNI toxicity. The patients' age at time of transplant, race, gender, and cause of ESRD were recorded (if known). The type of donor, induction regimen, incidence of delayed graft function (DGF), and baseline immunosuppression were recorded. Length of time between renal biopsy and conversion to belatacept was recorded, along with length of time between renal transplant and conversion to belatacept. The conversion protocol involved infusion of belatacept 10 mg/kg on days 1, 15; then 5 mg/kg on days 29, 43, and 58; then 5 mg/kg every 28 days thereafter.

The primary outcome of interest was postconversion eGFR. Values for eGFR were collected at multiple time points using the medical record: at 18 months, 12 months, and 6 months before conversion; at the time of conversion; and at 3, 6, 12, and 24 months after conversion. The eGFRs were estimated using the Modification of Diet in Renal Disease equation. Conversion eGFR values were obtained within 60 days of initiation of belatacept.

Secondary outcomes included (1) findings on renal biopsies preconversion, (2) the incidence of infections in each patient after conversion, and (3) the incidence of acute rejection one year after conversion. Renal biopsies were reviewed and scored by a renal pathologist, and chronic allograft damage index (CADI) scores were calculated. The CADI score is a composite of the Banff scores and includes pathological lesions commonly seen in transplanted kidneys [9, 11, 12]. Infections that were deemed significant included urinary tract infections (UTIs), pneumonias, cytomegalovirus (CMV) and parvovirus infections, and bacteremia.

Paired two-tailed *t*-tests were utilized to compare eGFR values at the time of conversion with eGFRs at 3, 6, 12, and 24 months after conversion. All statistical analyses were conducted using R version 3.0.1 (The R Foundation for Statistical Computing). A significance level of 0.05 was utilized for all statistical tests.

## 3. Results

Thirty-three patients who underwent renal transplantation between 1994 and 2015 were converted to belatacept at our institution. We excluded any patients who had not undergone renal biopsy prior to conversion to belatacept. Twenty-three patients had indication biopsies prior to belatacept conversion. Of these, 16 patients were excluded because they had findings other than IF/TA and arteriolar hyalinosis on biopsy (e.g., acute tubular necrosis, thrombotic microangiopathy). Seven remaining patients who had evidence of chronic CNI toxicity on biopsy were included in the study. These patients underwent transplantation between 1994 and 2007 and were converted to belatacept between 2010 and 2015.

The baseline characteristics of the 7 patients are shown in Table 1, including underlying causes of ESRD. All patients received a living donor transplant except for 1 patient. None of the patients had delayed graft function. 5 patients were induced with thymoglobulin (data was unavailable for patients 3 and 7, who were transplanted elsewhere). All patients were EBV IgG seropositive prior to conversion.

Table 2 shows the time between transplant and renal biopsy, the time between biopsy and conversion, and the time between transplant and conversion. The average time between transplant and conversion was 4,341 days or 11.9 years. There was variability with regard to the time between biopsy and conversion, ranging from less than 1 month to greater than 1 year after biopsy.

There was no decrease in eGFR at 3, 6, 12, or 24 months after conversion as compared with before conversion. The mean eGFR at time of preconversion was 32.9 mL/min, as compared with 35.6 mL/min at 3 months (*p* = 0.09), 34.1 mL/min at 6 months (*p* = 0.63), 34.9 mL/min at 12 months (*p* = 0.57), and 39.6 mL/min at 24 months after conversion (*p* = 0.92) (Table 3). The *p* values represent comparisons between the eGFR at conversion and eGFR at 3, 6, 12, and 24 months after conversion. Patients 2, 3, 6, and 7 had nonsignificant increases in their eGFRs during the 12 months after conversion; at 24 months after conversion, patients 3 and 7 continued to show improvements in their eGFR, while patient 6 had an improvement of 3 mL/min. Data was not available for patient 5 at 24 months after conversion because the patient was deceased from a myocardial infarction. Patient 1 eventually had loss of allograft after 12 months and was retransplanted.

Table 3 also shows the composite CADI scores and the extent of circumferential arteriolar hyalinosis. Patient 1 had the lowest CADI score of 3. The remainder of the patients had CADI scores greater than 4. None of the patients had evidence of cellular or acute/chronic humoral rejection. Though the biopsies were not uniformly stained for SV40 to look for evidence of BK virus, all patients had negative serum BK viral screens (with the exception of patient 5, who was not screened).

Four patients suffered from infections after conversion. One patient developed* E. coli* bacteremia with urosepsis, another suffered from an Enterococcal UTI, and a third had a* Citrobacter freundii* UTI. The fourth patient (patient 5) had an episode of* Campylobacter* gastroenteritis. All grafts survived except in the case of patient 1, who developed chronic graft dysfunction, not requiring hemodialysis. The patient was ultimately retransplanted 13 months after conversion.

There were no instances of acute rejection in the first year after conversion. Postconversion donor specific antibodies (DSA) were negative in all of the patients except for patients 5 and 6, in whom this was not reported. In patients 1, 3, and 4, the preconversion DSA was negative, whereas in patients 2 and 7, a preconversion DSA was not checked.

## 4. Discussion

This study of patients who underwent late conversion to belatacept (at least 7.7 years after transplant) suggested that belatacept may stabilize eGFR in patients who were initially on CNIs for maintenance immunosuppression. We found that 4 patients had an increase in eGFR from the time of conversion to 24 months after conversion (Table 3). While one patient (patient 5) did not have an increase in eGFR from the time of conversion to 12 months after conversion, there was a slight increase in eGFR from 1 year before conversion to 1 year after conversion (28 mL/min to 31 mL/min). Across all 7 patients, mean eGFRs increased at 24 months after conversion, increasing from 32.9 mL/min at the time of conversion to 39.6 mL/min at month +24. Belatacept conversion led to stabilization of eGFR at all time points, with a trend towards increased eGFR at 3 months.

Most belatacept conversion studies feature patients who have been converted much earlier in their posttransplant course as compared to our study. Wojciechowski et al. recently conducted a retrospective analysis of 20 patients converted to belatacept for prolonged delayed graft function, and the mean eGFR rose from 16 mL/min/1.73 m^2^ to 43 mL/min/1.73 m^2^ at 30 days after conversion [13].

Fewer studies have examined late conversion to belatacept. Brakemeier et al. looked at 69 transplant patients who had CNI-induced nephrotoxicity and were switched to belatacept at a mean of 69 months [8]. Mean eGFR increased from 26 ml/min/1.73 m^2^ to 34 ml/min/1.73 m^2^ at 12 months after conversion. The Kaplan-Meier estimates for patient and graft survival at 12 months were 95.0% and 85.6%, respectively. In this study, however, not all of the included patients were biopsied, and of those who were, not all of them had biopsy-confirmed CNI-induced nephrotoxicity.

The patients in our study were all biopsied at 7.7 to 16.8 years after transplant (Table 2). The time between biopsy and conversion was highly variable, with 2 patients converted within 1 month of biopsy, and others more than 1 year later (patients 3 and 7). This may have related to provider preference and the use of alternate strategies prior to belatacept conversion. The late conversion after biopsy may account for some of the lack of improvement in some patients.

With regard to biopsy findings, there was no overt correlation between CADI scores and eGFR. The chronic allograft damage index (CADI) score after transplantation assigns a numeric score of the extent of chronic injury in an allograft. Studies suggest that a score > 4 is associated with an increased risk of allograft loss at 3 years [12, 14]. Remarkably, all patients except for one had a score > 4; patient 1 had a CADI score of 3, yet had the lowest eGFR both before and after conversion, and ultimately suffered from allograft loss. The reasons for this are unclear, though this may be related to a history of recurrent UTIs requiring multiple courses of antibiotics, which may have contributed to graft dysfunction and the need for eventual retransplantation. Conversely, patient 3 had a high CADI score at 8, yet had an eGFR of 38 mL/min before conversion with a rise to 55 mL/min after conversion. There is debate about whether serum creatinine and eGFR are adequate predictors of graft loss, and CADI has therefore been extensively studied in experimental model as a “biomarker” of allograft function [10, 11]. Small sample size of patients may have limited any direct correlations between CADI scores, eGFR, and graft survival.

Belatacept was relatively well tolerated among our cohort. The most common infectious complication occurring in the year after conversion appeared to be UTIs, congruent with findings from Vincenti et al. [15]. None of the patients in our cohort developed CMV or BK viremia. The presence of infection after conversion may suggest the need to modify the conversion protocol to include lower immunosuppression in low risk patients.

All renal allografts were preserved at 1 year. There were no instances of rejection in this cohort, and there was no evidence of subclinical rejection on biopsy in any of the patients. Patient 1 was converted to belatacept when eGFR was already low at 20 mL/min. Despite transitioning to belatacept, renal function continued to deteriorate; the patient was eventually retransplanted shortly after 1 year after converting to belatacept.

There were several limitations to our study. We had a small sample size, thereby limiting the statistical significance and generalizability of our findings. Furthermore, for some patients, data related to cold ischemic time and HLA profiles/sensitization before transplant was unavailable. The biopsies were initially interpreted by a number of different pathologists, and when the results were ultimately compiled for this study, one pathologist interpreted the findings. The follow-up time for our study was relatively short, and belatacept may be more effective in preserving eGFR beyond 1 year after conversion. It is possible that any improvement in eGFR may simply have reflected reductions in CNI toxicity, with improvement in vasospasm and vasoconstriction after cessation of CNI therapy. Furthermore, many factors can influence eGFR, making data interpretation in a small study cohort challenging. Finally, it is possible that the natural history of these patients was such that eGFR may have stabilized with time irrespective of conversion to belatacept; perhaps inclusion of a control population in a larger study cohort could help address this question.

In summary, belatacept may be a safe and effective alternative to CNIs, as it may stabilize eGFR. Further investigation of conversion therapy for chronic CNI toxicity is warranted in order to determine which subsets of patients would most benefit from belatacept-based regimens.

## Figures and Tables

**Table 1:** Patient characteristics.

Patient	Age at transplant (years)	Gender	Race	Cause of ESRD	Donor type	Induction
1	37	Female	White	Nephrocalcinosis	Living related	Thymoglobulin
2	33	Female	White	Dysplastic kidney disease	Living related	Thymoglobulin
3	40	Male	White	Focal segmental glomerulosclerosis	Living related	Unknown
4	58	Male	White	Obstructive uropathy/chemotherapy	Living related	Thymoglobulin
5	50	Male	White	Lithium toxicity	Living unrelated	Thymoglobulin
6	44	Male	Asian	IgA nephropathy	Deceased donor	Thymoglobulin
7	21	Female	White	Unknown	Living related	Unknown

**Table 2:** Time between transplant and biopsy, biopsy and conversion, and transplant and conversion.

Patient	Time between transplant and biopsy (days)	Time between biopsy and conversion (days)	Time between transplant and conversion (days)
1	4705	26	4731
2	2773	19	2792
3	3250	392	3795
4	3796	97	3893
5	4508	217	4725
6	3554	241	3795
7	6145	517	6662

**Table 3:** eGFR mL/min/1.73 m^2^ before conversion, at conversion (month 0), and after conversion with associated allograft biopsy findings^*∗*^.

	eGFR								CADI score	% A	% CI	% CT
	Month −18	Month −12	Month −6	Month 0	Month 3	Month 6	Month 12	Month 24				
Patient 1	22	20	21	14	15	11	11	--	3	25%	20%	20%
Patient 2	39	46	43	38	40	40	50	37	6	20%	10%	10%
Patient 3	50	38	40	38	46	50	50	55	8	53%	40%	40%
Patient 4	47	47	43	46	43	38	34	28	5	60%	15%	15%
Patient 5	29	28	33	35	37	32	31	---	6	80%	20%	20%
Patient 6	47	40	34	31	37	37	34	41	9	31%	30%	30%
Patient 7	45	30	28	28	31	31	34	37	6	58%	20%	15%

Mean ± SD	39.8 ± 10.6	35.6 ± 10.0	34.6 ± 8.2	32.9 ± 10.1	35.6 ± 10.3	34.1 ± 12.0	34.9 ± 13.2	39.6 ± 9.8				
*p* value^†^	---	---	---	---	0.09	0.63	0.57	0.92				

^†^Paired two-tailed *t*-test, comparing eGFR at conversion with eGFR at subsequent months. CADI: chronic allograft damage index; A: arteriolosclerosis; CI: interstitial fibrosis; CT: tubular atrophy; SD: standard deviation. ^*∗*^Biopsy data was collected before conversion in all cases (see text). *Note.* Month 24 data was not available for patient 5, who is deceased. Patient 1 suffered allograft loss after month 12 and was retransplanted.
